# The quinoline framework and related scaffolds in natural products with anti-*Leishmania* properties

**DOI:** 10.3389/fchem.2025.1571067

**Published:** 2025-04-25

**Authors:** Gloria Yaluff, Leídi Herrera, Miriam Soledad Rolón, Celeste Vega, Hugo Cerecetto

**Affiliations:** ^1^ Departamento de Medicina Tropical, Instituto de Investigaciones en Ciencias de la Salud-Universidad Nacional de Asunción, Asunción, Paraguay; ^2^ Departamento de Biología, Facultad de Ciencias Exactas y Naturales-Universidad Nacional de Asunción, Asunción, Paraguay; ^3^ Laboratorio de Biología de Vectores y Parásitos, Instituto de Zoología y Ecología Tropical, Universidad Central de Venezuela, Caracas, Venezuela; ^4^ Centro para el Desarrollo de la Investigación Científica (CEDIC), Asunción, Paraguay; ^5^ Grupo de Química Orgánica Medicinal, Instituto de Química Biológica, Facultad de Ciencias-Universidad de la República, Montevideo, Uruguay; ^6^ Área de Radiofarmacia, Centro de Investigaciones Nucleares, Facultad de Ciencias-Universidad de la República, Montevideo, Uruguay

**Keywords:** 2-substituted quinoline, furoquinoline, polyhydroquinoline, bisbenzylisoquinoline, quinolinone

## Abstract

For almost one hundred years, the quinoline heterocycle has been recognized as a privileged pharmacophore in anti-*Leishmania* agents. Early on, the action of compounds with this scaffold, found in some natural alkaloids, was tested against different *Leishmania* stages and strains. Different structural arrangements containing the quinoline framework have been described in different *in vitro* and *in vivo* anti-*Leishmania* alkaloids, namely, quinoline proper, isoquinoline, and quinolone, among others. In recent years, new quinoline derivatives isolated from nature have been described, in addition to having carried out in-depth *in vitro* and *in vivo* biological studies, as well as chemical modifications to obtain new leaders. This review updates the state of the art on naturally occurring quinolines and some synthetic derivatives to provide therapeutic tools and strategies to explore new drugs based on this chemosystem for the treatment of leishmaniasis.

## Introduction

Leishmaniasis is a zoonotic vector-borne disease caused by protozoan parasites of the genus *Leishmania* (Eukarya, Kinetoplastea), transmitted to animals and humans through the bite of infected female phlebotomine sandflies. The disease presents in three main forms: cutaneous leishmaniasis (CL), mucocutaneous leishmaniasis (MCL), and visceral leishmaniasis (VL) (Steverding, 2017). The stigmatization in patients with the visible nature of CL and MCL is a significant social and psychological issue, leading to discrimination, social exclusion, and emotional distress (Wenning et al., 2022). There are approximately 700,000 to 1 million new cases of leishmaniasis every year, with an estimated 20,000 to 40,000 deaths in 98 endemic countries (WHO, 2024). The burden of the disease is concentrated in areas of Africa, the Middle East, South America, and parts of Asia. Efforts are ongoing to improve surveillance, control, and treatment strategies, as well as to develop new interventions such as vaccines and innovative diagnostic tools. There is no vaccine available for use in humans, and control of the disease relies mainly on chemotherapy. Current drugs are outdated, toxic, and unaffordable for most populations, while increasing resistance and treatment failures challenge their efficacy (Monzote, 2009).

The quinoline heterocycle has been acknowledged as a favored pharmacophore in anti-*Leishmania* agents for nearly 10 decades (Karamchandani, 1927; Varma, 1927; DasGupta and Dikshit, 1929; Devi, 1929). Compounds containing this scaffold, which are present in many natural alkaloids with different structural motives, including quinoline proper, isoquinoline, and quinolone, have shown to be effective *in vitro* and *in vivo* against various *Leishmania* strains and stages early on. Recent years have seen the description of novel quinoline secondary metabolites derived from primary metabolites discovered in nature, as well as extensive *in vitro* and *in vivo* biological research and chemical modifications, generating chemodiversity by using moieties not present in nature, to produce new anti-*Leishmania* leads. In the next section, we expose some recent aspects related to the investigation of new anti-*Leishmania* drugs based on this chemosystem.

## Historical background

**FIGURE 1:**
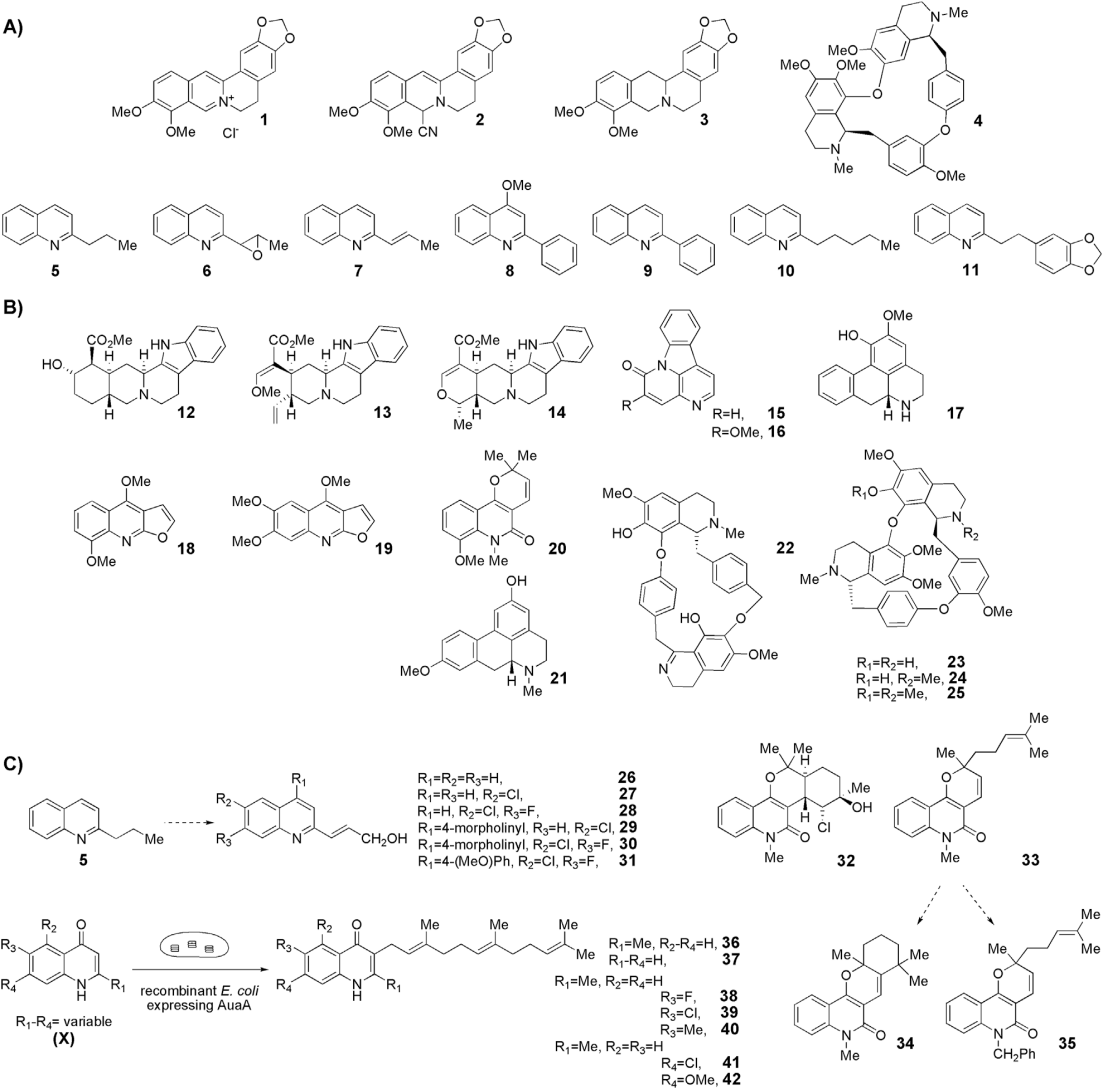
Some relevant natural quinolines with anti-*Leishmania* activities isolated **(A)** in the 20th century and **(B)** in the 21st century. Recent synthetic compounds with anti-*Leishmania* activities based on natural quinolines **(C)**.

In the 20th century, the first descriptions of naturally occurring quinolines date to the 1920s. Thus, berberine was one of the first alkaloids of the isoquinoline group, an ammonium salt with an interesting structure (**1**, Figure 1A), described as anti-*Leishmania* (Karamchandani, 1927; Varma, 1927). In the early 1990s, this alkaloid, along with other derivatives (natural and synthetic), was evaluated *in vivo* in golden hamster models of *Leishmania donovani* and *Leishmania braziliensis panamensis*, finding that it was less potent in *L. donovani* and more toxic (measuring weight loss) than the reference drug, Glucantime (Vennerstrom et al., 1990). Two synthetic derivatives (**2** and **3**, Figure 1A) emerged, due to their activities or toxicities, as possible leaders to consider in future studies.

Since the 1970s, Fournet et al. described a series of isolations of different quinolines that were studied *in vitro* and *in vivo* against *Leishmania*. In this sense, the bisbenzyltetrahydroisoquinoline isotetrandrine (**4**, Figure 1A), isolated from the roots, stems, leaves, and fruits of *Limaciopsis loangensis* Engl., a liana growing in west central tropical Africa to Angola (Cavé et al., 1979), was evaluated *in vitro* (Fournet et al., 1988) and *in vivo* with promising results (at 100 mg/kg/day), being better than the reference drug (Glucantime, at 200 mg/kg/day), against *Leishmania amazonensis* (PH8 and H-142) and *Leishmania venezuelensis* (VE/74/PM-H3) in Balb/c mice (Fournet et al., 1993a). In 1994, petroleum ether and chloroformic extraction of alkaloidal extracts of stem, leaves, and roots of *Galipea longiflora* Krause, a tree growing in Bolivia, Brazil, and Perú, were analyzed *in vitro* and *in vivo* against *Leishmania* spp. (Fournet et al., 1994b; Fournet et al., 1994c). The bioguided extract fractionations and purifications revealed 12 active alkaloids, 2-substituted quinolines **5–11**, with **5–8** (Figure 1A) having the most *in vitro* anti-promastigotes activity against different strains of *Leishmania braziliensis*, *L. amazonensis* and *L. donovani* (Fournet et al., 1994a) (IC_90_ in the range of 25–100 μg/mL with IC_90_ of 1 μg/mL for pentamidine and >100 μg/mL for Glucantime in all the studied strains). The authors did not mention alkaloids with no biological activity.

## The most recent naturally isolated quinolines and derivatives with leishmanicidal activities

In the 21st century, new alkaloids derived from quinoline framework with very varied and more complex structures were isolated and evaluated biologically, *in vitro* and *in vivo*, against *Leishmania*. Thus, in 2000, Staerk et al. (2000) described some alkaloids isolated from the bark of *Corynanthe pachyceras* K.Schum., a lower-storey forest tree growing in tropical West Africa, that were evaluated against different biosystems. Among the characterized compounds, perhydroisoquinoline **12** (Figure 1B) stood out for its *in vitro* activity against *Leishmania major* promastigotes (IC_50_ = 23.4 µM), nearly 10 times greater than the reference Pentostam (IC_50_ = 219 µM). The hexahydro-1*H*-quinolizine **13** (Figure 1B), related to **12**, was another secondary metabolite extracted from this botanical species that emerged as excellent anti-*Leishmania* agent (IC_50_ = 1.12 µM) together with the alkaloid **14** (ajmalicine, IC_50_ = 0.57 µM, Figure 1B), previously extracted from other botanical species, which is structural and metabolically related to this system but was not evaluated against this protozoan (Wei, 1965).

Since 2002, Ferreira et al. (2002) described a series of isolations of new quinoline derivatives that were studied *in vitro* and *in vivo* as potential anti-*Leishmania* drugs. From the stem bark of *Zanthoxylum chiloperone* var. *angustifolium* (Engl.), a tree growing in central and southern South America, they isolated a pair of azaquinolines, canthin-6-one, **15**, and 5-methoxycanthin-6-one, **16** (Figure 1B). These alkaloids were the two most active major constituents of the extract of *Z. chiloperone* stem bark *in vitro*. In another study from 2015, the authors studied the best harvesting time and seasonal dynamics to obtain the best yield of **16** from *Z. chiloperone* leaves (Cebrián-Torrejón et al., 2015). Using information from traditional medicine, Ferreira et al. screened different Paraguayan plants for anti-*Leishmania* activities and found that the crude extract of *Ocotea lancifolia* stem bark, a tree growing in Argentina, Brazil, Bolivia, and Paraguay, displayed relevant behaviors against three strains of promastigote of *L. braziliensis*, *L. amazonensis* and *L. donovani* (Fournet et al., 2007). Among the active alkaloids, the study identified **17** ((−)-cadaverine, Figure 1B), which also displayed a discrete selectivity index (SI), IC_50,mammal_/IC_50,protozoan_, of 1.6, using HepG2 hepatic cell line. Finally, Ferreira et al. (2010) isolated from the stem bark of *Helietta apiculata* Benth., a shrub or tree growing in South America (Argentina, Brazil, and Paraguay), a series of secondary metabolites and evaluated *in vitro* against *L. amazonensis*, *Leishmania infantum*, and *L. braziliensis* promastigotes and *in vivo* in a model of *L. amazonensis* infection in Balb/c mice. Among these metabolites, they found the furoquinoline **18** (γ-fagarine, Figure 1B) (Ahsan et al., 1994) as the most active alkaloid *in vitro* (IC_50_ between 17.3 and 26.5 µM), with higher activity than the chloroformic extract of *Helietta apiculata* (IC_50_ between 28.5 µM and 39.4 µM) but with much lower activity than the *in vitro* references (miltefosine, IC_50_ between 7.5 and 10.4 µM, and amphotericin B, IC_50_ between 0.01 and 0.05 µM). At that time, Coy Barrera et al. (2011) also described the isolation of furoquinolines, among other secondary metabolites, from stem bark or leaves of *Raputia heptaphylla* Pittier, a shrub or tree growing in the wet tropical locations of Bolivia, Colombia, Costa Rica, Panamá, Perú, and Venezuela. However, the furoquinolines (for example, **19**, Figure 1B) displayed poor *in vitro* activities against promastigotes and amastigotes of *Leishmania (V.) panamensis.* From this botanic species, the 2-quinolinone **20** (Figure 1B) emerged as a promising anti-promastigotes agent (IC_50_ = 52.7 µM, with IC_50_ = 2.5 µM for pentamidine and IC_50_ > 1098 µM for sodium stibogluconate).

In 2012, Mollataghi et al. (2012) described the isolation of a new alkaloid (**21**, Figure 1B), among others previously described in other botanical species, from the acidic percolate of the bark of *Beilschmiedia alloiophylla* (Rusby) Kosterm, a tree growing in Colombia, Costa Rica, Ecuador, Panamá, and Venezuela. This polyhydroquinoline, a methylated positional isomer of **17**, was the best anti-*Leishmania* alkaloid evaluated in this study (IC_50_ = 10.5 µM with an IC_50_ = 0.4 µM for miltefosine), although, unfortunately, the authors did not specify which strain and stage of the protozoan were used.

da Silva et al. (2012) evaluated the previously isolated bisbenzylisoquinoline warifteine (**22**, Figure 1B) against promastigotes of *Leishmania chagasi in vitro*. This alkaloid, purified from leaves of *Cissampelos sympodialis* Eichl., a climbing shrub growing in Brazil, was 50 times more active (IC_50_ = 135 µM) than the reference Glucantime. Regrettably, **22** had cytotoxicity against mammalian cells HEp-2 and NCI-H292 with SIs less than 1. Similarly, Naman et al. (2015), in a study that involved *in vitro* screening against *L. donovani* promastigotes of 234 isolated natural products, identified three bisbenzyltetrahydroisoquinolines (**23**, **24**, and **25**, Figure 1B) with protozoan IC_50_ lower than 10 µM. These alkaloids, which were purified previously from roots of *Thalictrum alpinum*, a perennial herb growing in the arctic and alpine regions of North America and Eurasia, displayed different SIs (using human colorectal cancer HT-29 cells as mammalian system) depending on the *N*- and *O*-methylation at SI_**23**_ = 29.3; SI_**24**_ = 3.6; and SI_**25**_ = 0.5.

### Some recent structural modifications based on the naturally isolated quinolines in the search for leishmanicidal agents

*Leishmaniasis* is not an exception to the long-standing practice of employing natural products as structural templates to produce novel bioactive compounds. In this regard, the active isolated 2-substituted quinolines (for example, **5**, Figure 1A) (Fournet et al., 1994a) were used as a structural reference to generate new synthetic derivatives. In 2003, Fakhfakh et al. (2003) synthesized approximately 50 quinolines by modifying them mainly in heterocycle position 2. In general, the 2-alkenyl derivatives displayed good anti-parasite activities, and the authors highlighted synthetic-quinoline **26** (Figure 1C) for its double antileishmanial and antiviral activity (Fakhfakh et al., 2003) that could be proposed as a drug for coinfection of HIV-*Leishmania*. A few years later, Gopinath et al. (2013), with the help of the Drug for Neglected Diseases initiative (DNDi, 2024), prepared some quinolines by modifying position 2 but also investigating modifications at positions 4, 5, 6, and 7 (for example, **27**, **28**, **29**, **30**, and **31**, Figure 1C). Approximately 25 quinolines with such characteristics were reported for their *in vitro* activities against intramacrophagic *L. donovani* amastigotes and against the Vero cell line (mammalian kidney epithelial cells), with descriptions of their different activities and selectivity profiles. 2,4,6,7-Tetrasubstitutedquinoline **30** emerged as the most selective of all the studied compounds, some of which had the following order: SI_**30**_ = 187.5; SI_**27**_ = 33.59; SI_**31**_ = 22.26; SI_**29**_ = 10.65; SI_**miltefosine**_ = 6.27; SI_**28**_ = 5.54; SI_**26**_ = 3.17; and SI_**5**_<1.25. Additionally, **30**, together with **26**, **31**, and miltefosine, was studied in an *in vivo* model of VL (Table 1). The *in vivo* results, together with some relevant results of **30** pharmacokinetic studies (Gopinath et al., 2014), indicate it could be a drug candidate to be investigated further.

**TABLE 1:** Summary of anti-*Leishmania* studies on animal models of natural quinolines and synthetic inspired by natural ones.

Compound	Source	*In vivo* model	Dosage	Results/Comments	References
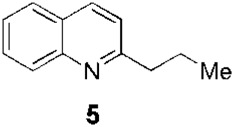	*G. longiflora* Krause	Balb/c mice infected with *L. donovani* (MHOM/ET/67/L82; LV9)	- Daily intraperitoneal, subcutaneous, or oral at 0.20 mmol/kg, 0.54 mmol/kg, or 0.70 mmol/kg for 5 or 10 days - Control: Glucantime (with the same administration regimen but at 0.54 mmol/kg)	Very good activity by the three routes of administration, although less active than Glucantime	Fournet et al. (1994c)
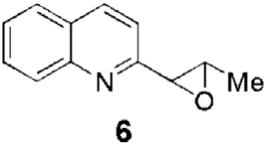					
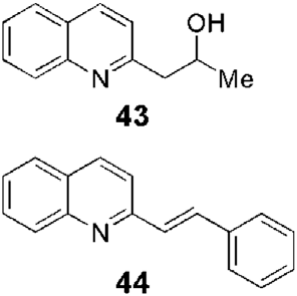	Synthetic			Poor *in vivo* biological performance	
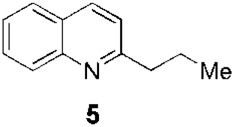	*G. longiflora* Krause	See text	See text	Different studies (using different administration regimens and different vehiculizations/salts, in combination with other drugs) were performed. Parasitic burden reduction in the liver (Nakayama et al., 2005) - For quinoline **5** (at 12.5 mg/kg): 66% - For miltefosine (at 7.5 mg/kg): 72%	Nakayama et al. (2005); Balaraman et al. (2015a); Balaraman et al. (2015b); Campos Vieira et al. (2011)
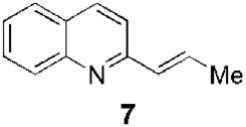	*G. longiflora* Krause	Balb/c mice infected with *L. amazonensis* MHOM/IFLA/BR/67/PH8 or *L. venezuelensis* MHOM/VE/74/PM-H3	- Twice daily orally at 50 mg/kg for 15 days or five times by intralesional injection - Control: Glucantime (with the same administration regimen but at 56 mg/kg, 28 mg/kg, or 14 mg/kg of Sb(V) for subcutaneous route or at 28 mg/kg of Sb(V) for five intralesional injections)	Oral administration at 50 mg/kg (*L. amazonensis*) - Decreased lesion weight: 70% - Decreased parasite load: 95% Intralesional administration at 50 mg/kg (*L. amazonensis*) - Decreased lesion weight: 74% - Decreased parasite load: 90% Parasite burden-reduction for control (at 28 mg/kg) - Subcutaneously: 95% - Intralesional injections: 96%	Fournet et al. (1996)
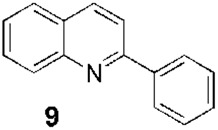	*G. longiflora* Krause	Balb/c mice infected with *L. amazonensis* IFLA/BR/67/PH8 or MHOM/GF/84/CAY-H-142	- Daily subcutaneous injections at 100 mg/kg for 14 days - Control: Glucantime (with the same administration regimen but at 56 mg/kg)	- As potent as control against MHOM/GF/84/CAY-H-142 strain - Less active than control against the virulent IFLA/BR/67/PH8	Fournet et al. (1994a)
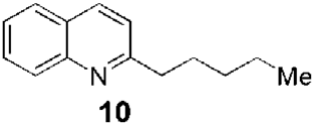				- Inactive	
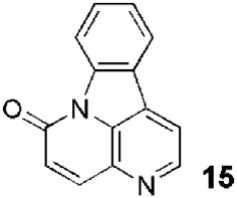	*Z. chiloperone* var. *angustifolium* (Engl.)	Balb/c mice infected with *L. amazonensis* MHOM/IFLA/BR/67/PH8	- Daily oral or intralesional route, at 10 mg/kg, for 15 days or four times, respectively - Control: Glucantime (subcutaneous injections for 10 days or five intralesional injections, both at 28 mg/kg of Sb(V))	Intralesional administration - Decreased lesion weight: 15% - Decreased parasite load: 77.6% Control - Decreased lesion weight: 28.5% - Decreased parasite load: 90.9%	Ferreira et al. (2002)
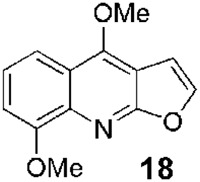	*H. apiculata* Benth	Balb/c mice infected with *L. amazonensis* MHOM/IFLA/BR/67/PH8	- Orally at 10 mg/kg for 15 days - Control: Glucantime (subcutaneous injections for 15 days at 100 mg/kg)	−3.4 times smaller lesion weight and 1.02 times higher percentage of suppression of parasite burden in lesions, both with respect to Glucantime treatment	Ferreira et al. (2010)
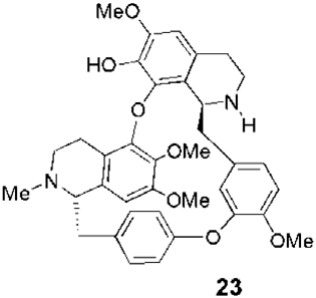	*T. alpinum*	Balb/c mice infected with *L. donovani* VL 82	- Intravenous injection at 2.8 mg/kg, 5.6 mg/kg, or 11.1 mg/kg for 2 weeks - Control: sodium stibogluconate (with the same administration but at 70 mg/kg)	- Parasitic burden reduction in spleen and liver cells, without toxicity signs, in a dose-dependent manner - Control: no significant decrease in parasitic burden in the spleen, while in the liver, the reduction was similar to that of **23**	Naman et al. (2015)
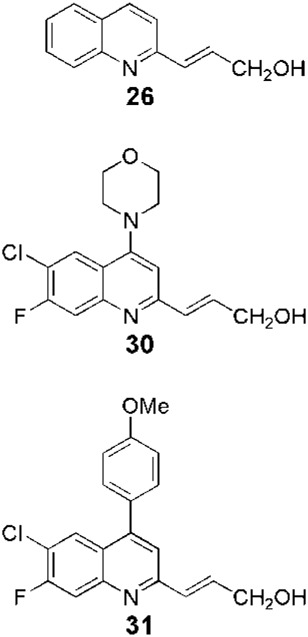	Synthetic	Inbred golden hamsters infected with *L. donovani* MHOM/IN/80/Dd8	- Once or twice daily intraperitoneal or oral administration at 50 mg/kg for 5 days - Control: miltefosine (with the same administration regimen but at 30 mg/kg)	- Parent quinoline **26** and tetrasubstituted quinoline **31** were inactive - The hydrochloride salt of **30**, orally and twice daily, was as active as miltefosine	Gopinath et al. (2013)
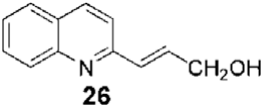	Synthetic	Balb/c mice infected with *L. donovani* MHOM/ET/1967/L82-LV9	- Daily oral administration at 25 mg/kg or 12.5 mg/kg for 10 days - Control: miltefosine (with the same administration but at 7.5 mg/kg)	Parasitic burden reduction in the liver - For quinoline **26** (at 25 mg/kg): 61% - For miltefosine: 72%	Nakayama et al. (2005)

In the preparation of the quinolone chloroaustralasine A (**32**, Figure 1C) Jézéquel et al. (2022) generated, as a synthetic intermediate, zanthosimuline (**33**, Figure 1C) (Wu and Chen, 1993) and other related subproducts with different structural motives, for example, **34** (Figure 1C). The authors synthesized **32** in order to know whether the anti-*Leishmania* activity previously described in the whole bark extract of *Codiaeum peltatum* (Billo et al., 2005) could be due to this quinolone. Additionally, from the *N-*demethylated **33,** the authors produced different *N-*substituted derivatives (for example, **35**, Figure 1C). The synthetic products **34** and **35** were active against intramacrophagic amastigotes of *L. infantum* and especially, unlike the parent compounds, selective toward the parasite, using RAW 264.7 macrophages as the mammal model; SI_**miltefosine**_ = 8.1; SI_**34**_ > 6.8; SI_**35**_ > 4.2; SI_**32**_ < 1.6; and SI_**33**_ < 0.8.

In 2023, Kruth et al. (2023), taking as a structural template the antibiotic isolated from different bacteria (Kunze et al., 1987), aurachin D (**36**, Figure 1C), described the preparation of a series of derivatives of it to be evaluated as anti-*Leishmania* agents using whole-cell biotransformations with recombinant *E. coli*. The *E*. *coli*, able to regioselectively incorporate the farnesyl moiety at the 3-position of the heterocycle by expressing aurachin farnesyltransferase AuaA (Kruth et al., 2022), was fed with quinolone precursors with different substitutions at 2-, 5-, 6- and/or 7-positions (**(X)**, Figure 1C). Of the 13 analyzed substrates, only three non-natural ones could not be biotransformed. The generated bio-products were evaluated *in vitro* against axenic amastigotes of *L. donovani,* and the selectivity was determined using rat myoblast L6 cells. 4-Quinolones **36** and **38** (Figure 1C) displayed better activities against the parasite than miltefosine, and some of the SIs were: SI_**36**_ = 2969.5; SI_**38**_ = 203.9; SI_**39**_ = 18.9; SI_**37**_ = 13.2; SI_**41**_ = 5.9; SI_**42**_ = 4.7; and SI_**40**_ = 4.3.

## Naturally isolated quinolines moving from lead to drug stage in the leishmanicidal pipeline

As a way of advancing in the anti-*Leishmania* quinoline-based drug pipeline, some *in vivo* efficacy, pharmacokinetic, safety, stability, formulation, and potential mechanisms of action studies have been carried out. These studies will be discussed below.

Fournet et al. (1994) conducted one of the most relevant analyses of the *in vivo* efficacy of natural quinolines. These authors evaluated 2-substituted quinolines **5** and **6** (Figure 1A) together with two synthetic derivatives, **43** and **44** (Table 1), finding that the natural ones were active against CL and VL (Fournet et al., 1994c) but less effective than the reference drug (Table 1). In another *in vivo* study (Fournet et al., 1994a), the main products of the alkaloidal extracts of *G. longiflora* Krause, that is, **9** and **10** (Figure 1A), were evaluated in models of CL. The authors found some interesting activity for **9** (Table 1). Two years later, the authors described the *in vivo* evaluation of the pure alkaloids **5**-**7** and **9–11** (Figure 1A) and two total alkaloidal extracts of *G. longiflora* Krause against a model of CL (Fournet et al., 1996). The results showed that **7**, with biological behavior similar to Glucantime (Table 1), is a leading compound to develop into oral anti-*Leishmania* therapy.

Continuing with the work on the pharmacological activity of bioactive anti-*Leishmania* secondary metabolites, Fournet and collaborators described *in vivo* studies and a model of CL using **15** and **16** (Figure 1B) (Ferreira et al., 2002). The best activity was observed for the azaquinoline **15** in an intralesional dose; however, it was discrete compared to Glucantime (Table 1). The furoquinoline **18** was also studied *in vivo* in the CL model by Fournet´s collaborators in 2010 (Ferreira et al., 2010), who found better activity at lower doses than Glucantime (Table 1).

The *in vitro* selective **23**, identified by Naman et al. (2015) as an anti-*Leishmania* agent, was tested *in vivo* in a murine model of VL. This bisisoquinoline displayed good activity in doses lower than Glucantime (Table 1).

In 1998, Iglarz et al. (1998) developed chromatographic methods using HPLC to determine natural-quinoline **5** (Figure 1A) in plasma and liver homogenates of mice. They found a hepatic distribution, where the parasites are localized, with an estimated half-life of 100 min, while in plasma, a second peak at approximately 4 h post-dosage appeared, indicating a possible enterohepatic cycle, although the liver results did not support this hypothesis. In 2005, Nakayama et al. (2005) studied the *in vivo* efficacy of some natural and synthetic 2-substituted quinolines on the CL, *L. amazonensis*, and VL, *L. infantum* and *L. donovani*, in order to obtain pharmacological data. The alkaloid **5** and synthetic **26** (Figure 1C) reduced the *L. donovani* parasite burdens in the liver with values similar to miltefosine (Table 1). After that, Campos Vieira et al. (2008) selected three 2-substituted quinolines, that is, **5** (Figure 1A), **26** (Figure 1C), and the *O*-methyl derivative of **26**, for study of their stability and acute oral toxicity. The alkaloid **5**, studied in methanol, DMSO, aqueous solution pH 7 (PBS), an aqueous solution of HCl pH 2, and a mixture of carboxymethylcellulose (0.5% in glucose 5%) and Tween 80 (0.5%) (CMC/T80) stored, was the most stable in all the cases, at 4°C or at room temperature with and without light. In the acute oral toxicity studies, administering a single dose of 10 mg/kg, 100 mg/kg, or 1000 mg/kg of quinoline **5** in the CMC/T80 emulsion and analyzing apparent toxicity signs and biochemical and hematological data, the authors found reversible toxicity signs (lethargic and staggering mice) only at 1000 mg/kg, a dose 80 times higher than the therapeutic dose found by Nakayama et al. (2005), without detected signs at the other doses.

The oily character of natural-alkaloid **5**, which affects its administration and bioavailability, has led to the study of different formulations to enhance its aqueous solubility. On the one hand, Campos Vieira et al. (2011) transformed **5** in its camphorsulfonic salt, **5-camp**, and found that **5-camp** retained the therapeutic efficacy of the free-base **5** and had comparable efficacy that miltefosine in the same dosage protocol, orally and daily at 60 μmol/kg of body weight for ten consecutive days.

Balaraman et al. tested two different approaches, in one case as a liposomal formulation and, in the other, as a hydroxypropyl β-cyclodextrin one. The liposomal vehiculization of **5** was active *in vitro* and *in vivo* in VL, intravenously administered at 3 mg/kg/day for 5 days (Balaraman et al., 2015a) and, in combination, at 0.75 mg/kg/day, with amphotericin B, at 6 μg/kg/day, for 5 days, and displayed a notable synergistic effect, which is, according the authors, a crucial factor in overcoming drug resistance. The formulation with the cyclodextrin was more than five times as active *in vitro* against intramacrophagic amastigotes of *L. donovani* than **5** alone and was also effective against resistant strains without inducing resistance (Balaraman et al., 2015b). In the *in vivo* studies, the intravenous administration of this formulation of alkaloid **5**, at 10 mg/kg/day for 10 days, reduced the parasite load in a comparable manner to miltefosine, without any evidence of toxicity. The pharmacokinetic studies performed by these authors found a rapid plasma concentration decline of **5** with a half-life of 58.7 min, which indicated that this formulation was *in vivo* effective for its adequate distribution throughout the tissues, facilitating the eradication of parasites in cases of disseminated leishmaniasis.

Some relevant mode and mechanism of action studies were described for some natural quinolines. Ghosh et al. (1985) demonstrated that amastigotes treated with the alkaloid **1** had (Figure 1A): i) inhibited respiration; ii) inhibited deoxyglucose uptake, indicating altered membrane composition; iii) interfered macromolecular biosynthesis. Additionally, **1** was able to interact with promastigote nuclear DNA *in vitro* and was found to be DNA-intercalating. Saha et al. demonstrated that **1**: i) triggered caspase-independent apoptosis-like death in promastigotes (Saha et al., 2009); ii) killed amastigotes by increasing NO production within the host macrophage (Saha et al., 2011); iii) and generated oxidative burst in infected neutrophils triggering apoptosis and in infected macrophages modulated mitogen-activated protein kinases (MAPKs).

Another pair of very interesting works by Calla-Magariños et al. found that the *G. longiflora* Krause extract, mainly composed of 2-substituted quinolines **5**, **8–11** (Figure 1A) with **9** being the most abundant, showed immunomodulatory activity on the host. The extract interfered with the activation of both mouse and human T cells, evidenced by the *in vitro* cellular proliferation reduction and interferon-gamma production (Calla-Magariños et al., 2009). Additionally, they found, unlike Glucantime, that the extract’s effect on the interferon-gamma was also seen *in vivo* with the concomitant IL-12 and tumor necrosis factor alpha suppression in restimulated spleen cells from total *Leishmania* lysate immunized animals (Calla-Magariños et al., 2013). These results showed that these quinolines would add a relevant advantage in the pharmacological treatment control of the chronic inflammatory reaction characterized in *Leishmania* infection, in addition to the anti-*Leishmania* activity.

The mechanisms of action and cellular deaths of natural quinolone **20** (Figure 1B) were studied, together with two synthetic quinolines, by Torres Suarez et al. (2020). The alkaloid **20** increased NO production in human peripheral blood (monocyte)-derived macrophages (MDMs) infected with *L. (V.) panamensis* promastigotes and produced necrosis-related ultrastructural alterations in intracellular amastigotes. On the other hand, the studied synthetic quinolines displayed different biological phenomena stimulating, in one case, oxidative breakdown in hMDMs and causing ultrastructural alterations in the parasite 4 h posttreatment and, in the other case, failing to induce macrophage modulation and selectively inducing apoptosis of infected hMDMs and alterations in the intracellular parasite ultrastructure.

## Concluding remarks

Even though few examples of natural quinolines and structurally related compounds tested against *Leishmania* have been described, the most promising skeleton for the anti-*Leishmania* therapies development is the bisbenzyltetrahydroisoquinoline system. Derivatives of this highly fused-cycle and functionalized system were shown to be effective in murine models of both CL and VL. Additionally, 2-substituted quinolines have been widely studied with models of CL and VL, both *in vitro* and *in vivo*, and should continue to be studied with different administration regimens or in combination with other agents. These studies could position these quinolines in a relevant place.

Regrettably, the pharmaceutical industry has not kept up with these studies, possibly because the compounds identified in these published findings cannot be patented, which has not allowed these agents to become “bench to bedside” entities.

In addition, unfortunately, biological studies have not yet been oriented to define whether there is a preference on the part of the quinoline derivatives against a certain type of *Leishmania* parasites that produce CL, MCL, or VL. Similarly, there have been few studies of the mechanism of action of natural quinolines that allow guiding the development of new quinoline-derived anti-*Leishmania* agents. Research should be directed in these fields.
